# Combatting the rising costs of cancer drugs; interventions from a university hospital’s perspective

**DOI:** 10.3389/fphar.2023.1264951

**Published:** 2023-08-28

**Authors:** Aniek Dane, Roelof van Leeuwen, Maaike Hoedemakers, Hugo van der Kuy, Stefan Sleijfer

**Affiliations:** ^1^ Department of Hospital Pharmacy, Erasmus MC, Rotterdam, Netherlands; ^2^ Department of Market Strategy and Healthcare Financing, Erasmus MC, Rotterdam, Netherlands; ^3^ Executive Board, Erasmus MC, Rotterdam, Netherlands

**Keywords:** cancer drugs, drug life cycle, university hospital, precision dosing, biomarkers, efficiency, sustainable healthcare, self-manufacturing

## Abstract

Rapid increase in cost continues to have negative impact on patients’ accessibility to life-changing anticancer medications. Moreover, the rising cost does not equate to similar increase in medication effectiveness. We recognise our responsibility as a university hospital to tackle this imbalance and strive to provide high quality, sustainable, affordable and accessible care. An active approach in cost containment of expensive and innovative cancer drugs was adopted in our organisation to safeguard accessibility and improve quality of life for patients. In this article, we described four inverventions: 1) identify right patient and minimise overtreatment, 2) in-house medicine production for selected indications, 3) minimise medicine spillages and 4) effective procurement strategies. We call on other hospitals to take action and, favourably, to collaborate on a European level. Together, we will safeguard the current and future care of our patients.

## 1 Introduction

We live in prosperous times when it comes to cancer care (Sleijfer and Verweij, 2016). Thanks to the advance of science and technology patients have better outcomes and quality of life following their diagnosis, even for rare indications.

According to the Dutch national figures of the last 30 years, 10-year survival rates have increased from 43% (1991–2000 period) to 59% (2011–2020 period)^1^. Meanwhile, cancer incidence increased from 58,505 patients in 1991 to 124,109* in 2022 (*preliminary figures)^2^ which is at a slower pace compared to the increasing costs of cancer drugs (Hofmarcher et al., 2020). The expenditure on reimbursed expensive medications in hospitals has increased from €1.24 billion in 2012 to €2.64 billion in 2021, with 50% of this amount being attributed to cancer treatments^3^ (SiRM, 2022).

As a result of improved outcomes, better diagnostics, increasing incidence and more and expensive treatment options, total spending on cancer care is rising posing a potential threat to the accessibility of these drugs (Brouwer et al., 2019; NZa, 2022).

Pharmaceutical companies justify the higher prices with reasons such as the costs of research & development (R&D) (Prasad and Mailankody, 2017; DiMasi and Pieters, 2018) and creating value for patients and society (Prasad et al., 2017; Picozzi et al., 2020). However, clinical benefit and costs of cancer treatment are not directly associated (Vokinger et al., 2020).

Currently, with advanced medicinal therapeutic products (ATMPs) such as gene and cell therapies entering the market at soaring prices, new pricing models need to be developed to safeguard patient access and prevent unjustified public funding. To accelerate patient access to innovative medicines and to manage the increase in drug expenditure, governments, policy makers, reimbursement agencies and health insurers, often in collaboration with the pharmaceutical industry, have developed several tools such as managed entry agreements, external reference pricing, Health Technology Assessment (HTA), and (international) horizon scanning of new drugs and extensions of indications coming to market. As stated in the Pharmaceutical Strategy for Europe of the European Commission, solutions along the entire drug life cycle should be considered as it offers a more comprehensive and integrated approach to address the challenge of rising drug expenditure. Furthermore, all stakeholders should be involved in tackling this problem (European Commission, 2020). In addition to current efforts, university hospitals should be more aware of the rising drug costs and be pro-active in taking matter in their hands.

## 2 University hospital’s social responsibility: vision of Erasmus MC

**FIGURE 1:**
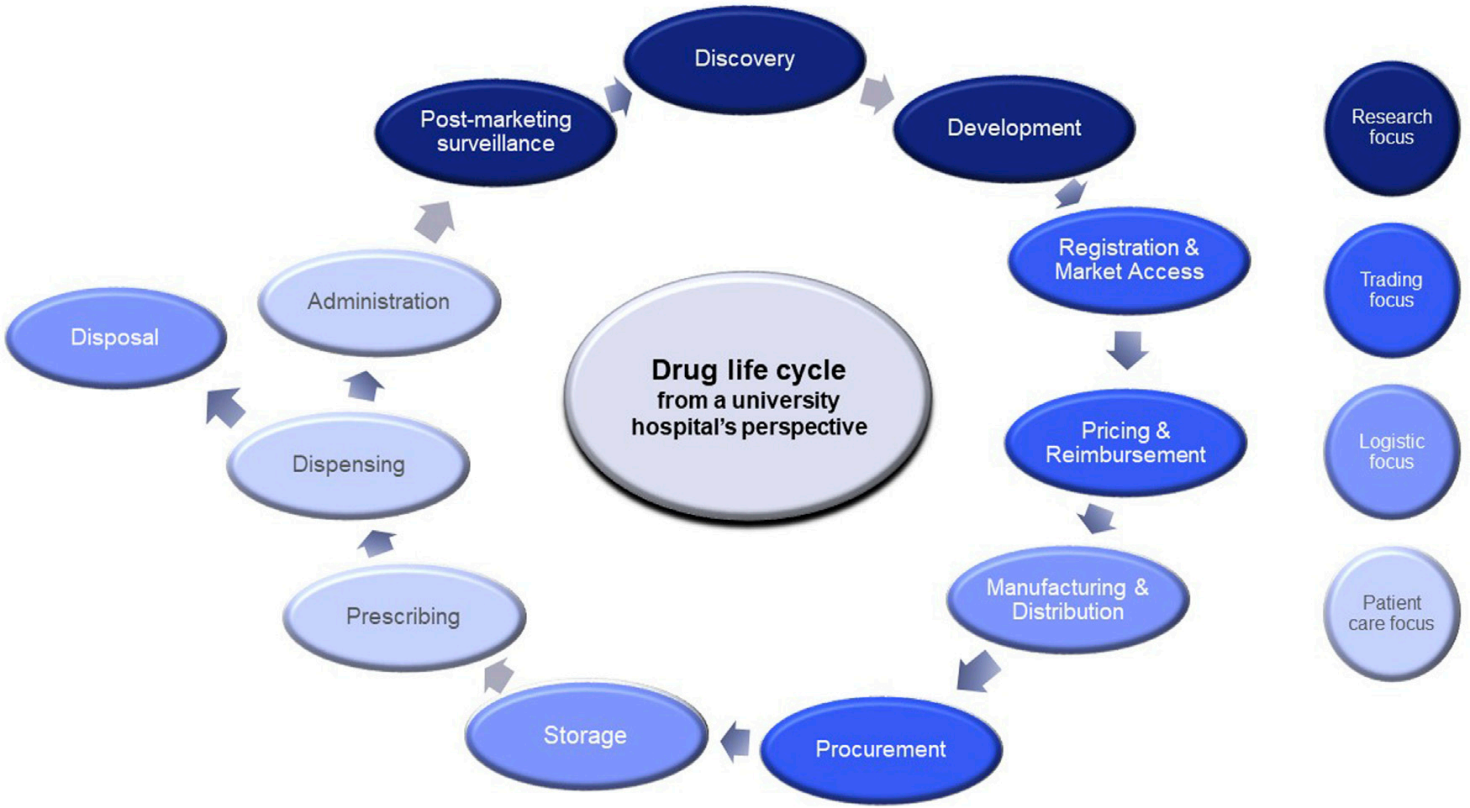
Drug life cycle from a university hospital’s perspective.

As one of the largest university hospitals in the Netherlands, we aim to contribute to a sustainable, affordable and accessible healthcare system. We have a responsibility to curb expenditure growth and ensure timely access to drugs for patients, while continue to improve patient care and quality of life. As university hospital, we are involved throughout the drug life cycle through healthcare delivery, education and academic research (see Figure 1).

Our involvement starts with conducting (bio) medical research leading to new anticancer drugs, then performing or coordinating clinical trials and collect real-world data for, e.g., post-marketing surveillance. In clinical settings, clinicians prescribe anticancer therapies while hospital pharmacy provides the medications or in some cases, manufactures them. Furthermore, we are involved in negotiating drug prices and discounts upon procurement. Here we described how our organisation delivers sustainable and affordable cancer care through several practical interventions.

### 2.1 Targeting the right patients

Before market entry of a new systemic anticancer therapy, the therapy must be rigorously assessed in a clinical trial, ideally with randomisation. Approval will be granted after the new therapy has demonstrated its benefits, preferably when the overall benefits, e.g., prolonging survival and/or improving quality of life outweigh the risks of toxicity and adverse drug reactions. To guide decisions on the value of novel treatments, criteria such as in the ESMO-Magnitude of Clinical Benefit Scale (ESMO-MCBS) (Cherny et al., 2017) have been developed which describes what magnitude of effect should be anticipated in the curative and non-curative setting.

In many clinical trials, participants are usually included based on stringent eligibility criteria such as tumour type, tumour stage, clinical performance, organ function, co-morbidity, presence of brain metastases, and prior treatments. These strict criteria unfortunately do not reflect the wider population. In clinical practice, criteria used to decide if a patient should receive a particular treatment are more lenient. An analysis in the Netherlands (van Zeijl et al., 2020) found that 40% of 2,536 systemically treated patients with advanced melanoma failed to meet the eligibility criteria used in the clinical trial. Quite often, patients included in clinical trials have better prognosis than the target population. For example, a study has reported that the median overall survivals between eligible and ineligible patients are 23 and 8.8 months respectively.

(van Zeijl et al., 2020). In many other tumour types and treatment regimens the same holds true. Worse outcomes in real-life populations are observed compared to the outcomes seen in the clinical trials due to less stringent selection of patients (Di Maio et al., 2019). Consequently, the benefit-risk ratio, which was deemed acceptable for patients accrued in the registration study, will not be met in the real-life populations. Therefore, many patients are exposed to toxicity from treatments that may not be outweighed by the benefits of the treatment. Likewise, expenditure to achieve the promised result is higher than reported during appraisal of the new therapy. In avoidance, treatments should be given only to patients who fulfil the same eligibility criteria of the clinical trial for that particular drug. Obviously, this is frequently not the case in practice. This underlines the necessity of aligning the eligibility criteria in clinical trials. By doing so, data collected in clinical trials will be applicable to a wider population which, successively, will reduce the risk of exposing patients to toxicity unnecessarily.

### 2.2 Clinical studies to reduce treatment burden for patients and to minimise costs of overtreatment

Overdiagnostics and overtreatment of cancer patients are very common and, though maybe inherent to cancer care, should be prevented where possible (Esserman and Thompson, 2013; Katz et al., 2018). The consequences of overtreatment are serious such as unnecessarily exposing patients to treatment toxicity and financial loss associated with overtreatment including medication administration, and adverse event management cost. There are several forms of overtreatment.

Many cancer patients display intrinsic resistance to treatment or develop resistance during treatment. It is important to detect failure to therapy as quickly as possible by scientific research and to swiftly implement effective therapies into daily clinical practice. Patients with intrinsic or primary resistant disease to a certain therapy can be identified by using predictive biomarkers. A great example is the identification of mutations in KRAS, NRAS, and BRAF as predictive markers in patients with metastatic colorectal cancer (mCRC) who are candidates for monoclonal antibodies (MoAbs) against EGFR (Sanchez-Ibarra et al., 2020). Initially all mCRC patients who failed the first line of chemotherapy were offered these agents. Subsequent research demonstrated that patients with tumours bearing the above mutations did not benefit from anti-EGFR MoAbs (Sartore-Bianchi et al., 2009). As a result, colorectal tumours are nowadays screened for the presence of these genetic variants and when present, patients are excluded from anti-EGFR MoAbs (ESMO guidelines). Evidently, there is a high clinical need for more of such predictive biomarkers. To find such biomarkers, the Centre for Personalized Cancer Treatment and the Hartwig Medical Foundation have initiated a nation-wide clinical study in which tumour biopsies were taken from metastases in patients with solid tumours, prior to starting a new line of treatment^4,^ ^5^ (Priestley et al., 2019). This has resulted in a large database with Whole Genome Sequencing data of metastases and outcome to treatments given, comprising data from almost 7,000 patients^6^. This database is accessible to researchers worldwide and will hopefully result in the discovery of other genetic markers with clinical utility.

Next to predictive markers, another important means to reduce overtreatment is detecting early markers of treatment failure (Smerage et al., 2014). Generally, in most cancer types, treatment is continued until objective progression is displayed by radiological assessments, which are often done at 2–3 months interval. Simpler methods executed at shorter time intervals can display resistance at an earlier time point by which overtreatment can be avoided. In this respect, liquid biopsies hold great promise and several studies are ongoing to assess their value as an early marker of response and to guide treatment.

Overtreatment does not limit to patients with failed therapy. It also affect patients who receivedurable benefit from treatment when the treatment intensity or treatment duration is higher than necessary to achieve the therapeutic aim. Evidence is somewhat lacking recommended dose intensity or treatment duration (e.g., number of cycles). This issue has long been recognized given many studies in the past, for example, comparing three *versus* four cycles of chemotherapy consisting of bleomycin, etoposide, cisplatin in patients with good-prognosis metastatic testicular cancer (de Wit et al., 2001) or shorter periods of adjuvant trastuzumab in primary breast cancer patients (Earl et al., 2019).

The same lack of evidence happens with costly new immunotherapies which are often administered until unacceptable toxicity or progression, whichever comes first, or for a maximum of 2 years. For example, monotherapy with MoAbs directed against PD-1 in advanced melanoma patients are given for 2 years without a strong rationale for this regimen. Several studies are ongoing to establish whether this treatment can be safely terminated in patients experiencing a confirmed response, which usually happens 6–9 months after treatment start, instead of prolonging therapy until 2 years consistent with the findings of clinical trials (Mulder et al., 2021). Another example is the SONIA study, in which CDK4/6 inhibitors have proven value when added to endocrine treatment in patients with hormone-receptor, HER2 negative metastatic breast cancer. It was, however, unknown whether CDK4/6 inhibitors should be applied in the first or the second line. In this randomised study, outcomes in terms of progression free survival and overall survival were similar between use in first line and second line. However, first line use was associated with 16.5 months longer treatment on CDK4/6 inhibitors, which led to a 42% increase in grade 3/4 toxicities and €180.000 higher costs per patient (Sonke et al., 2023). In other tumour types, similar studies are ongoing and although not all treatments can be shortened given the extensive heterogeneity in disease characteristics including treatment sensitivity, this is an important topic to explore (Lorigan and Eggermont, 2019; Waterhouse et al., 2020). Based on such studies, several guidelines already recommend shorter treatment periods (Keilholz et al., 2020; Sun et al., 2023).

### 2.3 Careful administration and avoidance of spillage: sustainable treatment and solid hospital finance

For convenience reasons, pharmaceutical companies have often justified their decision to change from weight-base dosing to fixed dose regimens. For example, pembrolizumab, in initial studies, a weight-base dose of 2 mg/kg/dose Q3W was demonstrated to be effective. This regimen was later adjusted to a fixed dose of 200 mg Q3W, which indicated there is no dose reduction recommended even if patient’s weight is lower than the average adult weight. However, it was demonstrated that weight-based dosing of pembrolizumab was equally effective and safe (Bayle et al., 2019). This published pharmacokinetic model (Bayle et al., 2019) has validated that a dosing regimen of 2 mg/kg Q3W could equate to 4 mg/kg/dose Q6W (with 400 mg as maximum dose) (Diekstra et al., 2020). Within our own clinical setting, as was described in Malmberg et al., the modified dose of 4 mg/kg Q6W with a maximum dose of 400 mg and a dose rounding margin of 10%, has not only provided effective and safe treatment for our patients, but also prevented potential overdose (Malmberg et al., 2022) and reduced costs by 22% (in 2022). This weight-based dose-capping strategy can be implemented for more expensive cancer treatments such as nivolumab and other immune checkpoint inhibitors (Hall et al., 2020).

Apart from efficient dosing, medicine spillage offers further cost saving benefits. Cancellation of intravenous (IV) therapy, which has been prepared in advance, due to toxicity of disease progression can lead to wastage. Therefore, Erasmus MC schedules patients undergoing the same treatment on the same day. In this way, when patients have to cancel on short notice, the therapy can be given to another patient. Another example, introduced by Radboud UMC in the Netherlands, is the reduction of spillage of oral oncology drugs. When patients with a progressive course of their disease stop their oral therapy, large quantities of oral anticancer drugs may be left unused. Collecting and re-dispensing these unused drugs can save money and contribute to achieve sustainability goals (Smale et al., 2021). Therefore, oncologists should find a balance between prescribing adequate medication supply for patients’ home and the risk of destroying unused surplus when therapy change is required.

### 2.4 Local production to save costs on trial medication and medical treatment

Many commercialised cancer drugs were discovered by researchers of university hospitals. The first prototypes of these potential drugs, used for preclinical testing, are often manufactured inside this hospital. For instance, in 2004, the first European clinical studies on solid tumours with CAR-T cells were performed at Erasmus MC with an in-house product (Lamers et al., 2006). The same goes for lutetium-labelled octreotide—Lu-177-DOTATATE –, a very effective radiopharmaceutical therapy for patients with gastroenteropancreatic neuroendocrine tumours (GEP-NET), which was discovered at Erasmus MC. For over 20 years it has been successfully administered to large groups of patients within the Netherlands and abroad (Strosberg et al., 2017). In 2017, this treatment was registered in Europe, after an industry-sponsored phase III study (Strosberg et al., 2017). In 2019, at market entry in the Netherlands, the drug price was substantially higher than our own manufacturing costs at that time. Negotiations between health insurers and the pharmaceutical company unfortunately resulted in the withdrawal of this treatment from the Dutch market in 2020. Currently, Erasmus MC produces this drug for its own patients,and, in 2022, reduced costs by 42% compared to the costs of the licensed product. Similarly, we recently started producing Lu-177-PSMA for patients with prostate specific membrane antigene (PSMA)- positive metastatic castration-resistant prostate cancer (mCRPC). We expect costs of the licensed product when it enters the Dutch market will be higher than our own pharmacy preparation.

Erasmus MC is not the only Dutch university hospital which makes effort to provide its patients with affordable medicines. Amsterdam UMC has started to produce chenodeoxycholic acid (CDCA) capsules through in-house production—a drug for treatment of the rare metabolic disorder cerebrotendinous xanthomatosis (CTX) which was prescribed off-label–, after the pharmaceutical company had registered this drug in 2017 and increased the price 500 fold (Polak et al., 2021). These examples demonstrate that university hospitals are capable of successfully manufacturing medicines for their patients at lower costs and should be further explored internationally.

### 2.5 Affordable pricing: smart procurement and novel pricing models

In the Netherlands, medicines can only be procured by hospitals after they have been granted market authorization at a national or European level and have obtained a reimbursement status. Generally, purchase prices are being established in negotiations between pharmaceutical companies and hospitals in which the former has an incentive to aim for the highest price in order to create shareholder value and the latter aims for the lowest price given a restricted healthcare budget. In an attempt to negotiate prices to the lowest possible level, the Dutch university hospitals cooperate in a joint procurement board^7^. However, focusing solely on the lowest price is not a sustainable solution as this strategy may reduce the number of pharmaceutical suppliers and therefore diminish competition to a, in the worst case, monopoly position. Moreover, having fewer suppliers put the supply chain at risk during potential medicine shortages. Focussing on price may be more favourable in the short term, but in the long run it is more beneficial to secure competition. In 2020, Erasmus MC, together with several health insurers, has decided to not always select the cheapest supplier in order to foster competition.

While negotiating prices is an effective way of lowering expenditure on pharmaceuticals, the real question is whether or not buyers are paying a fair price, i.e., being delivered value-for-money. Even though call for transparency on the costs of drugs increases, pharmaceutical companies are reluctant to disclose how prices are being established. In response to that, researchers from Erasmus University Rotterdam proposed and successfully applied a novel pricing model to calculate a fair price for oncology drugs which displays the cost-based prices (including R&D costs and profit margin) are substantially lower than the list price (Uyl-De Groot and Löwenberg, 2018; Thielen et al., 2022). Researchers from Amsterdam UMC created an alternative pricing model for establishing the price for a repurposed drug—mexiletine—using a recent European drug-pricing model^8^ as a framework to include actual costs incurred (van den Berg et al., 2021).

### 2.6 Future research and activities

We touched upon several successful interventions and we will continue to explore other interventions such as registration of in-house discovered and developed drugs, raising awareness on cost-effective prescribing, developing start and stop criteria, and research on less intense treatment schemes (e.g., lowering dose per administration, extending intervals between administrations, shortening treatment duration and boosting of medication).

Regarding clinical studies, researchers from university hospitals can compare the eligibility criteria in the study with patient characteristics in daily life and adapt them accordingly. Moreover, researchers and clinicians have access to real-world data that can be used in the reassessment of clinical efficacy and cost-effectiveness of cancer drugs after market approval. We will continue to expand our efforts to reduce spillage by optimizing shelf life of drugs in stock and pooling. Finally, we must discuss with our patients the possibility of non-treatment and focus on quality of life instead of quantity of life.

Most importantly, a strong collaboration between the hospital pharmacy department and medical departments is the key to successful intervention implementation.

We anticipate even more opportunities for (university) hospitals to combat the rising costs of cancer drugs, if we collaborate on a European level. We can start by sharing best practices on precision dosing and avoidance of spillage, collaborate on promising new technologies such as ATMPs and strive collectively for fair prices.

To overcome legal barriers, we should collaborate on adapting European legislation to be more open to self-manufacturing of medicines, and find innovative ways to jointly purchase expensive (cancer) drugs. Lastly, explore solutions—both on an European and national level–for legal issues regarding off-label prescribing (e.g., with precision dosing of pembrolizumab) is essential.

## 3 Conclusion

Our aim was to inspire other hospitals to take action. Here, we have focused on several strategies to prevent overtreatment and to minimise costs of expensive anti-cancer drugs such as clinical research on identification of predictive markers and, precision dosed administration of drugs, local manufacturing and effective procurement methods. These strategies illustrate potential contribution of (university) hospitals to the reduction of expenditure growth on cancer drugs, while maintaining access, effectiveness and safety.

We urge other hospitals to review their own activities throughout the drug life cycle, collaborate on overcoming barriers and help contributing to a sustainable and affordable healthcare system.

## Data Availability

The original contributions presented in the study are included in the article/Supplementary material, further inquiries can be directed to the corresponding author.

## Footnotes

1
https://applicatie.nkrcijfers.nl/?fs%7Cepidemiologie_id=527&fs%7Ctumor_id=1&fs%7Coverlevingssoort_id=532&fs%7Cperiode_van_diagnose_id=601%2C600%2C599%2C598%2C597%2C596&fs%7Cjaren_na_diagnose_id=688%2C689%2C690%2C691%2C692%2C693%2C694%2C695%2C696%2C697%2C698%2C699&cs%7Ctype=line&cs%7CxAxis=jaren_na_diagnose_id&cs%7Cseries=periode_van_diagnose_id&ts%7CrowDimensions=periode_van_diagnose_id&ts%7CcolumnDimensions=jaren_na_diagnose_id&lang%7Clanguage=nl.

2
https://nkr-cijfers.iknl.nl/#/viewer/9cdbca8b-084c-4ab9-8ed5-12f719441877.

3
https://www.vektis.nl/intelligence/publicaties/factsheet-dure-geneesmiddelen.

4
https://www.hartwigmedicalfoundation.nl/.

5
https://www.cpct.nl/.

6
https://www.hartwigmedicalfoundation.nl/en/research-and-science/onderzoek/.

7
Inkoopcombinatie Ziekenhuis Apotheken Academische Ziekenhuizen (IZAAZ) NFU.

8 AIM European Fair Price Calculator for Medicines: Home: Fair Pricing Calculator.
